# Perceived weight stigma among adults diagnosed with schizophrenia in China: a cross-sectional study

**DOI:** 10.3389/fpsyg.2026.1928934

**Published:** 2026-09-22

**Authors:** Jiayuan Peng, Huan Wen, Jianjian Wang, Ka Yan Ho, Xiangmin Tan, Ting Pan, Mei Sun

**Affiliations:** 1 Xiangyin Campus of Xiangxing College, Hunan University of Chinese Medicine, Yueyang, China; 2 The First Affiliated Hospital of Xiamen University, Xiamen, China; 3 Clinical Nursing Teaching and Research Section, The Second Xiangya Hospital of Central South University, Changsha, China; 4 Department of Psychiatry, and National Clinical Research Center for Mental Disorders, The Second Xiangya Hospital of Central South University, Changsha, China; 5 School of Nursing, The Hong Kong Polytechnic University, Hung Hom, China; 6 School of Rural Health, Monash University, Warragul, VIC, Australia; 7 School of Nursing, Changsha Medical University, Changsha, Hunan, China; 8 Xiangya School of Nursing, Central South University, Changsha, Hunan, China

**Keywords:** coping style, perceived weight stigma, psychological distress, schizophrenia, social support, weight bias internalisation

## Abstract

**Background:**

Perceived weight stigma is increasingly recognised as an important psychosocial stressor associated with adverse psychological well-being, impaired healthcare engagement, and poorer treatment outcomes. Adults diagnosed with schizophrenia may be especially vulnerable because of antipsychotic-related weight gain and the intersection of weight stigma and mental illness stigma. However, evidence regarding perceived weight stigma in this population remains scarce, particularly in low- and middle-income countries.

**Objective:**

To characterise perceived weight stigma and identify factors associated with it among adults diagnosed with schizophrenia in China.

**Methods:**

In this cross-sectional study, 301 adults diagnosed with schizophrenia completed standardized self-report measures assessing perceived weight stigma, weight bias internalisation, psychological distress, social support, coping styles, and sociodemographic characteristics. A generalized linear model with a negative binomial distribution and log-link function was used to identify factors associated with perceived weight stigma.

**Results:**

Overall, 54.2% of participants reported perceived weight stigma, operationalised as endorsement of at least one PWSQ item (PWSQ score ≥1). Lower monthly income, higher weight bias internalisation and greater psychological distress were associated with higher perceived weight stigma. BMI was not significantly associated with perceived weight stigma.

**Conclusion:**

This study provides quantitative evidence on perceived weight stigma, an under-researched psychosocial issue among adults diagnosed with schizophrenia. In this Chinese clinical sample, perceived weight stigma was common and was associated with lower income, greater weight bias internalisation, and greater psychological distress. These findings highlight that perceived weight stigma is a clinically relevant psychosocial phenomenon. Shifting from symptom-focused psychiatric care to a broader social psychology determinants framework may be essential for supporting more comprehensive recovery-oriented interventions within this population.

## 1 Introduction

Weight stigma is the social devaluation, stereotyping, or discriminatory treatment of individuals based on their body weight (National Eating Disorders Association, 2023). Weight stigma can affect individuals across the weight spectrum, although it is reported more frequently among individuals with higher body weight (Brewis et al., 2018; Prunty et al., 2020; Puhl et al., 2021). Mounting evidence indicates that weight stigma is associated with a wide range of adverse psychological, behavioural, and physical outcomes, including depression, medication non-adherence, weight gain, reduced healthcare utilisation, poorer treatment adherence, and even increased risks of suicide and mortality (Daly et al., 2020; Puhl et al., 2013; Richardson et al., 2014; Rodriguez et al., 2019; Schvey et al., 2019; Sutin et al., 2015). Furthermore, weight stigma may undermine the quality of healthcare by reducing trust in healthcare providers and contributing to inequitable healthcare delivery (Gudzune et al., 2014; Phelan et al., 2015b). Weight stigma can manifest in multiple forms, including experienced weight stigma (e.g., overt discriminatory behaviours), perceived weight stigma (i.e., the perception that one is judged or treated negatively because of body weight), internalised weight stigma (i.e., self-directed endorsement of negative weight-related stereotypes), and anticipated weight stigma (i.e., expecting future discrimination based on body weight) (Alimoradi et al., 2020; Hunger et al., 2020).

Among these, perceived weight stigma reflects individuals’ subjective awareness and interpretation of weight-based prejudice and constitutes an important psychological pathway through which weight stigma influences health. Adults diagnosed with schizophrenia may be especially vulnerable to perceived weight stigma because they are situated at the intersection of multiple sources of disadvantage. Owing to antipsychotic medication side effects (World Health Organization, 2018), genetic predisposition, and socioenvironmental determinants (e.g., stigma, low socioeconomic status, and social isolation) (Manu et al., 2015), they are at substantially increased risk of overweight and obesity compared with the general population. Epidemiological evidence indicates that the prevalence of overweight or obesity among general adults population is approximately 52% (World Health Organization, 2021), whereas rates among adults diagnosed with schizophrenia may reach 60% (Annamalai et al., 2017). In addition, the increasing sociocultural idealization of thinness in China (Stojcic et al., 2020) may further heighten individuals’ sensitivity to body weight. Consistent with this, a qualitative study identified that medication-related weight gain led some individuals diagnosed with schizophrenia to perceive increased negative judgment from others based on their weight (Haracz et al., 2018). Furthermore, mental illness stigma is already highly prevalent among individuals diagnosed with schizophrenia (Dubreucq et al., 2021). The coexistence of mental illness stigma and weight-related stigma may heighten their sensitivity to weight-based social evaluation, making perceived weight stigma particularly salient among adults diagnosed with schizophrenia.

Understanding perceived weight stigma among adults diagnosed with schizophrenia is of substantial clinical and public health importance. Stigma has been identified as a major barrier to recovery and social reintegration among individuals receiving antipsychotic treatment (Weiss, 2006). Individuals diagnosed with schizophrenia have long experienced neglect and inequitable treatment due to mental illness stigma (Thornicroft et al., 2007), and the additional burden of weight stigma may further exacerbate these disadvantages. More importantly, improving the health and well-being of individuals diagnosed with mental illness through a social determinants of health framework has become an increasingly important priority in contemporary mental healthcare (Wand, 2024). The World Health Organization (WHO) and the Office of the United Nations High Commissioner for Human Rights (OHCHR) (World Health Organization, 2023) have emphasized that mental healthcare should move beyond a narrow focus on diagnosis, pharmacological treatment, and symptom reduction toward addressing the broader social determinants influencing mental health outcomes.
Despite its potential clinical significance, evidence on perceived weight stigma among adults diagnosed with schizophrenia remains limited. To our knowledge, only one quantitative study has examined weight stigma in this population. Conducted in the United States, that study assessed experienced and internalised weight stigma among individuals diagnosed with schizophrenia or schizoaffective disorder, but not perceived weight stigma (Barber et al., 2011). Perceived weight stigma therefore remains unexamined in this population, leaving an important gap in the literature.

Previous studies in the general population have identified several factors associated with perceived weight stigma, including BMI, socioeconomic characteristics, weight bias internalisation, psychological distress, coping styles, and social support (Alimoradi et al., 2020; Bidstrup et al., 2022b; Gerend et al., 2022; Hatzenbuehler et al., 2009; Phelan et al., 2015a). However, these potential correlates have not been comprehensively examined in this population.

Given the limited evidence on perceived weight stigma among adults diagnosed with schizophrenia this study investigated perceived weight stigma in a sample of Chinese adults diagnosed with schizophrenia. Specifically, we aimed to characterise perceived weight stigma and examine its correlates. By addressing this evidence gap, the study seeks to advance understanding of perceived weight stigma as a clinically relevant psychosocial phenomenon and to inform more comprehensive, recovery-oriented mental healthcare that extends beyond symptom management to encompass broader social determinants of mental health.

## 2 Methods

### 2.1 Design and participants

This cross-sectional study was conducted between June 2023 and January 2024. A convenience sampling strategy was used to recruit adults diagnosed with schizophrenia from inpatient wards of a tertiary psychiatric hospital in China. Prior to data collection, the research team obtained approval and support from the hospital’s psychiatric director and psychiatric head nurse and signed a formal agreement with the hospital that included a guarantee to keep patient information confidential.

Participants were screened and recruited with the assistance of nurses and physicians according to predefined inclusion and exclusion criteria. Researchers distributed paper questionnaires after giving a comprehensive explanation of the study objectives and procedures to the participants and obtaining their written informed consent. All questionnaires were completed independently by participants under standardized instructions provided by trained researchers. Completed questionnaires were collected on-site and reviewed for completeness. For missing, incorrect, or garbled items, participants were invited to correct or fill in on the spot based on the voluntary principle.

The inclusion criteria for participants were as follows: (1) diagnosed with schizophrenia based on the International Classification of Diseases 11th revision (ICD-11), (2) age ≥ 18 years, and (3) having cognitive and communicative capacity to participate in the study. Eligibility was assessed by psychiatrists and psychiatric nurses during routine clinical evaluation. Participants were required to be able to understand the study information and questionnaire instructions, communicate coherently with the research team, and complete the questionnaire independently. Individuals with dementia or intellectual disability that impaired their ability to cooperate with the investigation were excluded.

The initial sample size was estimated using the commonly applied rule of at least 10 participants per variable (Miller and Kunce, 1973). Based on 19 candidate variables, a minimum of 190 participants was required. Allowing for a 15% rate of incomplete or invalid responses, the target sample size was increased to 224 participants. A total of 315 questionnaires were initially collected. Fourteen questionnaires were not filled out completely (the rate of omission exceeded 15%) due to the participants’ withdrawal, which were considered invalid questionnaires. Finally, 301 valid questionnaires were obtained, and the efficiency of questionnaire collection was 95.6%.

### 2.2 Measures

#### 2.2.1 Demographic characteristics

Age, gender, ethnicity, marital status, educational level, residence, employment status, monthly income, weight before antipsychotic treatment, and duration of disease were self-reported. A portable height and weight measuring instrument was used to measure the participants’ current weight (kilograms) and height (meters). The BMI (kg/m^2^) was calculated using the standard formula of weight divided by height squared. Weight change after antipsychotic treatment was calculated by subtracting pre-antipsychotic body weight from current body weight.

#### 2.2.2 Perceived weight stigma questionnaire (PWSQ)

Perceived weight stigma was assessed using the PWSQ. The scale comprises 10 dichotomous items, each rated as yes (1) or no (0). Item scores are summed to yield a total score ranging from 0 to 10, with higher scores indicating higher levels of perceived weight stigma. For descriptive purposes, participants who endorsed at least one PWSQ item (PWSQ score ≥ 1) were classified as having reported perceived weight stigma. Similar operational definitions have also been adopted in previous studies (Crompvoets et al., 2024). In regression analyses, the total PWSQ score was retained as a continuous variable to reflect the cumulative burden and severity of perceived weight stigma. The Chinese version of the PWSQ demonstrated good internal consistency among Chinese university students (Cronbach’s alpha = 0.84) (Lin et al., 2020). In the present study, the PWSQ also demonstrated good internal consistency (Cronbach’s alpha = 0.84).

#### 2.2.3 Weight Bias Internalisation Scale (WBIS)

The WBIS consists of 11 items rated on a 5-point Likert scale ranging from 1 (strongly disagree) to 5 (strongly agree). After reverse scoring items 1 and 9, item scores were summed to obtain a total score, with higher scores indicating greater weight bias internalisation. The original English version of the WBIS demonstrated high internal consistency (Cronbach’s alpha = 0.90) (Durso and Latner, 2008). The Chinese version has also shown satisfactory psychometric properties among Chinese children and adolescents (Pakpour et al., 2019). In this study, the WBIS demonstrated high internal consistency with a Cronbach’s alpha of 0.90.

#### 2.2.4 Multidimensional scale of perceived social support (MSPSS)

Perceived social support was assessed using the MSPSS, developed by Zimet et al. (1990). The MSPSS evaluates perceived support from family, friends, and significant others using 12 items rated on a 7-point Likert scale ranging from strongly disagree (1 point) to strongly agree (7 points). Total scores ranged from 12 to 84, whereas subscale scores ranged from 4 to 28, with higher scores indicating greater perceived social support. The MPSSS has demonstrated good internal consistency among Chinese adolescents (total scale Cronbach’s alpha = 0.93; family support = 0.82; friend support = 0.88; and significant others support = 0.85) (Zhu, 2022). In this study, the Cronbach’s alpha for the total scale, family support subscale, friend support subscale, and significant others support subscale were 0.93, 0.89, 0.90, and 0.86, respectively.

#### 2.2.5 Simplified coping style questionnaire (SCSQ)

The scale consists of 20 items, including 12 items assessing positive coping and 8 items assessing negative coping (Xie, 1998). Items are rated on a 4-point Likert scale ranging from 0 (never) to 3 (very often). The SCSQ has been widely used in China and has demonstrated satisfactory internal consistency (Ma et al., 2014). In the present study, the Cronbach’s alpha for the total scale, positive coping subscale, and negative coping subscale were 0.85, 0.87, and 0.77, respectively.

#### 2.2.6 Distress thermometer (DT)

Psychological distress was assessed using the DT, developed by the National Comprehensive Cancer Network (NCCN) Distress Management Panel (Riba et al., 2019). The DT is an 11-point visual scale used to assess psychological distress levels over the past week. Scores range from 0 (no distress) to 10 (extreme distress). The Chinese version of the DT has demonstrated good reliability, with a test–retest reliability coefficient of 0.80 (Tang et al., 2011), and is recommended as a rapid screening tool for psychological distress (Donovan et al., 2014).

### 2.3 Analysis

All statistical analyses were conducted using IBM SPSS Statistics version 29.0 (IBM SPSS Inc., Chicago, IL, USA). Statistical significance set at a two-tailed *p* < 0.05. Categorical variables were described by frequency and percentages, while continuous variables were described by ranges, means, and standard deviations. Based on quantile-quantile plot and the Kolmogorov–Smirnov criteria, the total PWSQ score was non-normally distributed. Accordingly, the Mann–Whitney U test and Kruskal-Wallis test were used to examine group differences, and Spearman’s rank correlation was used to assess associations between variables.

To identify factors associated with perceived weight stigma, generalized linear models were applied. The PWSQ total score was a non-negative count variable with substantial overdispersion (mean = 2.17; variance = 7.421). A negative binomial model with a log link was therefore fitted. Approximately half of the participants had a PWSQ score of zero. A zero-inflated negative binomial (ZINB) model was also evaluated. The uncorrected and AIC-corrected Vuong tests favored the ZINB model (z = −4.46, *p* < 0.001; z = −2.90, *p* = 0.002, respectively). The BIC-corrected test showed no difference between the models (z = −0.01, *p* = 0.494). The more parsimonious negative binomial model was retained for the primary analysis.

Variables included in the multivariable analysis were selected based on the bivariate analyses and previous evidence on factors associated with perceived weight stigma. Variables significantly associated with PWSQ scores in the univariate or correlational analyses were included. BMI was additionally included because of its established association with weight stigma in previous studies.

### 2.4 Ethical considerations

This study was conducted according to the guidelines of the Declaration of Helsinki and approved by the Ethics Committee of the School of Nursing, Central South University (Approval No. E202382). Prior to participation, all participants provided written informed consent. All collected data were kept strictly confidential. Electronic data were encrypted and accessible only to authorized members of the research team.

## 3 Results

### 3.1 Descriptive analysis

As shown in Tables 1, 2, a total of 301 participants were included in the study, comprising 146 females and 155 males. The mean age of participants was 26.98 years (SD = 9.64), and the mean BMI was 23.55 kg/m^2^ (SD = 4.25). The maximum disease duration was 30 years, and the greatest weight gain after antipsychotic treatment was 42 kg. More than three-quarters of the participants were unmarried. Approximately 40.9% had attained a college degree or higher. Regarding employment status, most participants had never been employed (43.9%) or were jobless (34.9%).

**Table 1:** Descriptive analysis of categorical variables (*n* = 301).

Variables	Category	*n*	%
Gender	Male	155	51.5
	Female	146	48.5
Ethnicity	Han	275	91.4
	Minority	26	8.6
Marital status	Unmarried	230	76.4
	Married	47	15.6
	Divorced	24	8.0
Educational level	Junior high school or below	64	21.3
	High school	114	37.9
	College or above	123	40.9
Residence	Urban	143	47.5
	Rural	158	52.5
Employment status	Never employed	132	43.9
	Employed	56	18.6
	Jobless	105	34.9
	Retired	8	2.7
Monthly income (yuan)	< 3,000	82	27.2
	3,000–5,000	119	39.5
	> 5,000	100	33.2

**Table 2:** Descriptive analysis of continuous variables (*n* = 301).

Variables	Min	Max	Mean	SD
Age	18.0	71.0	26.98	9.64
BMI (kg/m^2^)	15.0	35.8	23.55	4.25
Disease duration (year)	0.1	30.0	6.35	5.91
Weight change after antipsychotic treatment (kg)	−21.0	42.0	7.17	8.31
PWSQ	0.0	10.0	2.17	2.72
WBIS	0.0	43.0	17.56	8.53
Family support	4.0	28.0	19.10	6.03
Friend support	4.0	28.0	18.07	5.83
Significant others support	4.0	28.0	19.17	5.62
SCSQ-A	0.0	36.0	22.10	7.54
SCSQ-P	0.0	24.0	11.07	5.29
DT	0.0	10.0	4.41	3.10

BMI, Body Mass Index; PWSQ, Perceived Weight Stigma Questionnaire; WBIS, Weight Bias Internalisation Scale; SCSQ-A, Active Coping of the Simplified Coping Style Questionnaire; SCSQ-P, Passive Coping of the Simplified Coping Style Questionnaire; DT, Distress Thermometer.

Overall, 54.2% of participants (n = 163) reported perceived weight stigma, operationalised as endorsement of at least one PWSQ item (PWSQ score ≥1) (see Figure 1). The mean PWSQ score was 2.17 (SD = 2.72).

**Figure 1:**
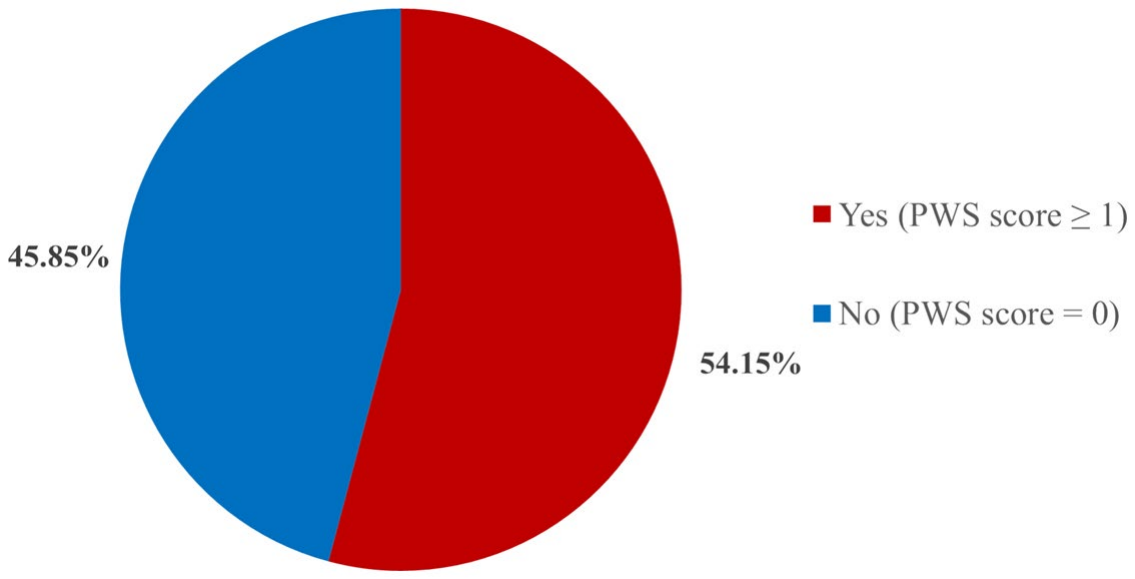
Proportion of participants reporting perceived weight stigma (*n* = 301).

### 3.2 Univariate analysis

As shown in Table 3, perceived weight stigma differed significantly among participants with different education levels (*p* = 0.028), residence (*p* = 0.048), and monthly income (*p* = 0.047). Participants living in rural areas had higher PWSQ scores than those living in urban areas (*p* = 0.048). Post-hoc pairwise comparisons showed that participants with a high school education had higher PWSQ scores than those with a college education or above (adjusted *p* = 0.030). Participants with a monthly income below RMB 3,000 had higher PWSQ scores than those earning more than RMB 5,000 (adjusted *p* = 0.041). No other pairwise differences were significant.

**Table 3:** Univariate analysis of perceived weight stigma (*n* = 301).

Variables	Categories	Median (interquartile range)	H/Z	*p*
Gender			−1.186	0.236
	Male	1.00 (3.00)		
	Female	1.00 (5.00)		
Ethnicity			−0.583	0.560
	Han	1.00 (4.00)		
	Minority	0.50 (3.50)		
Marital status			1.686	0.430
	Unmarried	1.00 (4.25)		
	Married	0.00 (3.00)		
	Divorced	1.50 (4.00)		
Educational level			7.185	0.028
	Junior high school or below	1.00 (5.00)		
	High school	1.00 (5.00)		
	College or above	0.00 (3.00)		
Residence			**−**1.976	0.048
	Urban	1.00 (3.00)		
	Rural	1.00 (5.00)		
Employment status			1.791	0.617
	Never employed	1.00 (5.00)		
	Employed	1.00 (3.00)		
	Jobless	1.00 (4.00)		
	Retired	0.00 (3.25)		
Monthly income (yuan)			6.134	0.047
	< 3,000	2.00 (5.00)		
	3,000–5,000	1.00 (4.00)		
	> 5,000	0.00 (3.00)		

*Post-hoc* pairwise comparisons were performed with Bonferroni correction. Significant differences were observed between the high school and college-or-above groups (adjusted *p* = 0.030) and between participants with monthly incomes < RMB 3,000 and > RMB 5,000 (adjusted *p* = 0.041).

### 3.3 Correlational analysis

Spearman correlational analysis showed that perceived weight stigma was positively correlated with weight bias internalisation (*r* = 0.545, *p* < 0.01), passive coping (*r* = 0.168, *p* < 0.01), psychological distress (*r* = 0.342, *p* < 0.01), and weight change after antipsychotic treatment (*r* = 0.177, *p* < 0.01). Conversely, perceived weight stigma was negatively correlated with family support (*r* = −0.144, *p* < 0.05), friend support (*r* = −0.234, *p* < 0.01), significant others support (*r* = −0.266, *p* < 0.01), and active coping (*r* = −0.131, *p* < 0.05) (see Table 4).

**Table 4:** Correlational analysis of perceived weight stigma (*n* = 301).

Variables	1	2	3	4	5	6	7	8	9	10	11	12
1. Age	1.000											
2. Disease duration (year)	0.587^**^	1.000										
3. BMI (kg/m^2^)	0.149^**^	0.200^**^	1.000									
4. Weight change after antipsychotic treatment (kg)	0.034	0.265^**^	0.579^**^	1.000								
5. WBIS	0.013	0.118^*^	0.252^**^	0.279^**^	1.000							
6. Family support	0.068	0.027	0.108	−0.017	−0.170^**^	1.000						
7. Friend support	0.041	0.021	0.007	−0.088	−0.193^**^	0.535^**^	1.000					
8. Significant others support	0.074	0.001	−0.010	−0.082	−0.281^**^	0.731^**^	0.589^**^	1.000				
9. SCSQ-A	0.107	0.025	0.009	−0.039	−0.190^**^	0.250^**^	0.342^**^	0.322^**^	1.000			
10. SCSQ-P	0.066	−0.029	0.139^*^	0.068	0.156^**^	−0.031	−0.008	−0.042	0.240^**^	1.000		
11. DT	−0.030	0.078	−0.010	0.082	0.277^**^	−0.180^**^	−0.164^**^	−0.120^*^	−0.159^**^	0.175^**^	1.000	
12. PWSQ	−0.066	0.073	0.110	0.177^**^	0.545^**^	−0.144^*^	−0.234^**^	−0.266^**^	−0.131^*^	0.168^**^	0.342^**^	1.000

^**^p < 0.01; ^*^p < 0.05.

BMI, Body Mass Index; WBIS, Weight Bias Internalisation Scale; SCSQ-A, Active Coping of the Simplified Coping Style Questionnaire; SCSQ-P, Passive Coping of the Simplified Coping Style Questionnaire; PWSQ, Perceived Weight Stigma Questionnaire; DT, Distress Thermometer.

### 3.4 Multivariate analysis

The negative binomial generalized linear model demonstrated that monthly income, weight bias internalisation, and psychological distress were significantly associated with perceived weight stigma (see Table 5).

**Table 5:** Multivariate analysis of perceived weight stigma (*n* = 301).

Variables	B	SE	exp(B)	95% CI for exp.(B)	Wald χ^2^	*p*
Educational level						
Junior high school or below	−0.295	0.238	0.745	0.468, 1.186	1.541	0.214
High school	−0.008	0.191	0.992	0.682, 1.442	0.002	0.967
College or above	Reference					
Residence						
Urban	−0.267	0.177	0.766	0.541, 1.083	2.280	0.131
Rural	Reference					
Monthly income (yuan)						
< 3,000	0.523	0.207	1.688	1.125, 2.533	6.386	0.012
3,000–5,000	0.443	0.195	1.557	1.062, 2.284	5.143	0.023
> 5,000	Reference					
BMI (kg/m^2^)	−0.029	0.025	0.971	0.926, 1.019	1.390	0.238
Weight change after antipsychotic treatment (kg)	0.010	0.012	1.010	0.988, 1.033	0.780	0.377
WBIS	0.093	0.012	1.097	1.072, 1.122	63.050	< 0.001
Family support	0.024	0.020	1.025	0.986, 1.065	1.577	0.209
Friend support	−0.009	0.017	0.991	0.959, 1.024	0.279	0.597
Significant others support	−0.037	0.021	0.963	0.924, 1.004	3.088	0.079
SCSQ-A	0.011	0.013	1.001	0.986, 1.037	0.692	0.405
SCSQ-P	0.013	0.017	1.013	0.980, 1.047	0.583	0.445
DT	0.096	0.026	1.100	1.045, 1.158	13.345	< 0.001

Model diagnostics indicated good fit (deviance/df = 1.000; Pearson χ^2^/df = 0.958). No substantial multicollinearity was observed (tolerance range: 0.332–0.906; VIF range: 1.103–3.016). The overall model was statistically significant (likelihood ratio χ^2^ = 145.009, df = 14, *p* < 0.001).

BMI, Body Mass Index; WBIS, Weight Bias Internalisation Scale; SCSQ-A, Active Coping of the Simplified Coping Style Questionnaire; SCSQ-P, Passive Coping of the Simplified Coping Style Questionnaire; DT, Distress Thermometer.

Compared with participants whose monthly income exceeded RMB 5,000, those earning less than RMB 3,000 (exp(B) = 1.688, *p* = 0.011) and those earning RMB 3,000–5,000 (exp(B) = 1.575, *p* = 0.020) reported significantly higher levels of perceived weight stigma. In addition, higher weight bias internalisation (exp(B) = 1.095, *p* < 0.001) and greater psychological distress (exp(B) = 1.101, *p* < 0.001) were associated with higher perceived weight stigma.

## 4 Discussion

The present study provides one of the first quantitative investigations of perceived weight stigma among adults diagnosed with schizophrenia in China. More than half of participants (54.2%) reported perceived weight stigma, as indicated by endorsement of at least one PWSQ item, suggesting that such psychological stressors were common in this clinical sample. The increasing sociocultural idealisation of thinness in China (Stojcic et al., 2020) may shape perceptions of weight-related social evaluation. As perceived weight stigma has not previously been examined specifically among people with schizophrenia, direct comparison with schizophrenia samples from other countries is not currently possible. Further quantitative studies across different cultural settings are needed to determine whether these findings are replicated in other populations diagnosed with schizophrenia.

The present study also extends the limited evidence by examining both psychological and socioeconomic correlates. Lower monthly income was associated with higher perceived weight stigma. This finding is consistent with sociological frameworks that situate stigma within unequal distributions of social and economic power (Link et al., 1989; Link and Phelan, 2001). Population-based data from England similarly showed higher odds of perceived weight discrimination among less wealthy adults (Jackson et al., 2015). Income may also reflect access to resources relevant to mental health care and weight management. In China, adults with schizophrenia from lower-income families were less likely to use pharmacological treatment and psychotherapy (Ding et al., 2022), while financial hardship was a common barrier to participation in weight-management programmes among people with serious mental illness and limited their ability to implement dietary and exercise recommendations (Olmos-Ochoa et al., 2019). Socioeconomic disadvantage may therefore be associated with both greater perceived exposure to weight-related devaluation and reduced access to supportive resources. These pathways were not assessed in the present study and warrant further investigation.

Perceived weight stigma was positively associated with psychological distress, consistent with previous evidence, consistent with previous evidence (Alimoradi et al., 2020). This finding highlights the close relationship between psychological functioning and perceived weight stigma. However, longitudinal evidence regarding their temporal relationship remains limited. In a one-year longitudinal study of young adults in Hong Kong, Fung et al. (2026) found that higher initial levels of perceived weight stigma were associated with higher initial levels of psychological distress, but changes in perceived weight stigma were not significantly associated with changes in psychological distress over time. Because the present study was cross-sectional, the temporal ordering of this association among adults diagnosed with schizophrenia cannot be established. Further longitudinal studies are therefore needed to establish the temporal ordering and direction of the association between perceived weight stigma and psychological distress in this population.

Weight bias internalisation was another important factor associated with perceived weight stigma among adults diagnosed with schizophrenia. This finding is consistent with evidence from other populations showing a positive association between perceived weight stigma and weight bias internalisation (Bidstrup et al., 2022a; Bidstrup et al., 2022b). Weight bias internalisation reflects the incorporation of societal weight-related biases into one’s self-concept (Alimoradi et al., 2020). Ratcliffe and Ellison (2015) proposed that exposure to weight stigma may contribute to the development and maintenance of weight bias internalisation, a pathway supported by subsequent longitudinal evidence (Keast et al., 2023; O'Brien et al., 2016). Conversely, their formulation model also suggests that individuals with greater weight bias internalisation may be more sensitive to weight-related social cues and may interpret ambiguous social interactions more negatively (Ratcliffe and Ellison, 2015). This may contribute to greater perceptions of stigma. These findings suggest that perceived weight stigma and weight bias internalisation may be closely interrelated. However, this association should be interpreted cautiously. The two constructs are conceptually related and were both assessed using self-report measures at the same time. Conceptual overlap and shared-method variance may therefore have contributed to the observed association. Longitudinal studies are needed to clarify the relationship between these constructs.

Another notable finding was the absence of a significant association between BMI and perceived weight stigma. This contrasts with previous evidence among adults living with obesity, where higher BMI was associated with greater perceived weight stigma (Crompvoets et al., 2024). Several contextual factors may be relevant to interpreting this divergence among adults diagnosed with schizophrenia. First, the co-occurrence of mental illness–related stigma and weight stigma may result in schizophrenia-related stereotypes, such as dangerousness and unpredictability, partially overshadowing the influence of body size in social evaluation (Waite et al., 2022). Second, from the perspective of health prioritization, individuals diagnosed with psychosis often prioritize psychiatric symptom stabilization and functional recovery over weight management, thereby reducing the salience of body weight in both self-perception and social appraisal (Waite et al., 2022). Finally, this population may be more likely to attribute weight gain to external and uncontrollable factors, particularly antipsychotic medication use, rather than to personal responsibility (Waite et al., 2022). Given that attributional processes play a central role in shaping sensitivity to weight stigma (Gerend et al., 2020), such external attribution patterns may weaken the psychological linkage between objective BMI and perceived weight stigma. Taken together, these findings suggest that perceived weight stigma may reflect broader psychosocial processes rather than objectively measured body weight alone. Future studies are needed to further examine the relative contributions and potential interactions among these factors.

## 5 Implications

The present findings have important implications for psychological science, clinical practice, and mental health policy. First, the frequent reporting of perceived weight stigma in this clinical sample highlights its potential relevance to the psychological care of adults diagnosed with schizophrenia. Routine psychological assessment may therefore benefit from considering patients’ perceptions of weight-related social evaluation alongside symptom severity and physical health.

Second, the associations of perceived weight stigma with weight bias internalisation and psychological distress identify potentially modifiable psychological processes that warrant further study. Longitudinal research is needed to establish their temporal relationships, followed by intervention studies to determine whether targeting internalised weight-related beliefs and psychological distress improves psychological outcomes.

Finally, the socioeconomic gradient observed in the present study highlights that perceived weight stigma is embedded within broader social and structural contexts rather than arising solely from individual body weight. Consistent with the growing emphasis on addressing the social determinants of mental health, these findings support a broader recovery-oriented model of psychiatric care in which social disadvantage, stigma, and psychological well-being are considered alongside symptom management. Future longitudinal and intervention studies are needed to determine whether changes in weight bias internalisation and psychological distress are accompanied by changes in perceived weight stigma and longer-term recovery outcomes among adults diagnosed with schizophrenia.

## 6 Limitations

This study has several limitations. First, the psychosocial measures and pre-antipsychotic body weigh were self-reported and may introduce the possibility of response biases such as social desirability bias and recall bias. Although the PWSQ, WBIS, and MSPSS have demonstrated acceptable psychometric properties in Chinese populations, they have not been specifically validated among adults diagnosed with schizophrenia. Their construct validity in this population therefore warrants further evaluation. Second, participants were recruited by convenience sampling from the inpatient wards of a single tertiary psychiatric hospital, and the sample was relatively young (mean age, 26.98 years). This may limit the generalisability of the findings to other regions, care settings, and populations with different socioeconomic or clinical characteristics. Future multicentre studies with more diverse samples are needed. Third, the cross-sectional design limits the ability to establish causal relationships between variables. Longitudinal or experimental studies are needed to clarify the direction and potential causal pathways of these associations.

Fourth, sample size was estimated using a general rule rather than a method specific to negative binomial regression. Model-specific calculations require assumptions about the outcome distribution, dispersion, and expected effect sizes, which were difficult to specify given the limited quantitative evidence in this population. Finally, several potentially important clinical confounders, including antipsychotic type and dose, treatment duration, symptom severity, hospitalization history, and metabolic comorbidities, were not assessed. This may have resulted in residual confounding. A more comprehensive investigation of these factors could enrich the understanding of perceived weight stigma among adults diagnosed with schizophrenia in China.

## 7 Conclusion

This study provides one of the first quantitative investigations of perceived weight stigma, an under-researched psychosocial issue among adults diagnosed with schizophrenia in China. More than half of participants reported perceived weight stigma. Lower monthly income, greater psychological distress, and higher weight bias internalisation were associated with higher perceived weight stigma, whereas BMI was not. These findings contribute evidence from a Chinese clinical sample to the emerging international literature on perceived weight stigma in schizophrenia. The observed associations also underscore the importance of psychosocial and socioeconomic context in understanding perceived weight stigma. Studies across culturally and clinically diverse populations are needed to assess the consistency of these associations and to inform culturally sensitive, person-centred, and recovery-oriented mental healthcare.

## Data Availability

The raw data supporting the conclusions of this article will be made available by the authors, without undue reservation.
